# Archives of Ophthalmology and Otology

**Published:** 1872-09

**Authors:** 


*Archives of Ophthalmology and Otology.* Vol. II. No. II. New York: W. Wood & Co., 1872.

This able Journal, of which Prof. Knapp, of New York, and Prof. Moos, of Heidelberg, are the accomplished editors, has, with this number, completed its second volume, and may be considered as established in its position among the medical periodicals of the day.

The Journal is too widely circulated and has been too long before the public to need an extended notice. We can simply say to our readers who are desirous of deeping pace with the advances being made in ophthalmology that this Journal is admirably suited to their wants.

Messrs. Wood & Co., the gentlemanly publishers, will be pleased to receive subscriptions. Their address is 27 Great Jones St., New York City.

